# A longitudinal study of psychological distress during and after COVID‐19 restrictions in caregivers of children with intellectual disability in the UK

**DOI:** 10.1002/jcv2.12261

**Published:** 2024-09-06

**Authors:** Karri Gillespie‐Smith, Karen Goodall, Doug McConachie, Jo Van Herwegen, Hayley Crawford, Carrie Ballantyne, Caroline Richards, Thomas Gallagher‐Mitchell, Joanna Moss, Grace Khawam, Laura Outhwaite, Emily Marriott, Freyja Steindorsdottir, Hope Christie

**Affiliations:** ^1^ Department of Health and Clinical Psychology School of Health in Social Science University of Edinburgh Edinburgh UK; ^2^ Institute of Education University College London London UK; ^3^ Division of Health Sciences Warwick Medical School Warwick University Coventry UK; ^4^ Division of Psychology School of Education and Social Science University of West of Scotland Paisley UK; ^5^ School of Psychology University of Birmingham Birmingham UK; ^6^ Department of Psychology Liverpool Hope University Liverpool UK; ^7^ School of Psychology University of Surrey Guildford UK

**Keywords:** coping, COVID‐19, intellectual disabilities, longitudinal, mental health

## Abstract

**Introduction:**

The current study explored longitudinally whether child behaviours that challenge and caregiver coping strategies was associated with psychological distress in caregivers of children with and without intellectual disability during and after lockdown.

**Method:**

An online survey was completed by caregivers who had children with and without intellectual disability during Time Period 1 (T1; August‐December 2021, *n* = 171) and then again during Time Period 2 (T2; January–March 2022, *n* = 109).

**Results:**

Child behaviours that challenge and caregiver psychological distress reduced in T2 compared to T1. Child behaviours that challenge, emotion focussed coping and avoidant coping was associated distress at both time points in caregivers of children with and without intellectual disabilities.

**Conclusions:**

The study shows that both child behaviours that challenge and caregiver psychological distress reduced as lockdown ended. However, caregiver coping strategies may have contributed to psychological distress, which has potential implications for interventions and support for caregivers.

**Key points**

- This is the first study to look at both child behaviours that challenge, caregiver coping strategies and caregiver distress in families with and without children who have intellectual disabilities during and after Covid‐19 lockdown in the UK.
- Caregivers showed higher levels of all coping behaviours (emotion focussed, problem focussed and avoidant) during Covid‐19 compared to after Covid‐19, with caregivers engaging in Avoidant coping least and more Emotion Focussed coping. Since maladaptive coping strategies (such as Emotion Focused and Avoidant) can lead to poor mental health outcomes, this indicates that caregiver coping would be a useful area to target for intervention during difficult times.
- Both caregivers of children with Intellectual Disabilities and caregivers of children without Intellectual Disabilities showed similarities in coping strategies during and after Covid‐19, however caregivers of children with Intellectual Disabilities showed higher levels of psychological distress compared to caregivers of typically developing children.
- Child behaviours that challenge, emotion focussed coping and avoidant coping was associated with psychological distress at both time points in caregivers of children with and without intellectual disabilities. This indicates a complex bi‐directional relationship between child behaviours and caregiver coping that impacts levels of caregiver psychological distress.

## INTRODUCTION

The first UK wide Coronavirus (COVID‐19) lockdown to reduce the spread of the virus (Cabinet Office, 2020) occurred from March to June 2020, followed by a second lockdown in Wales from October to November 2020, and in England and Northern Ireland from November to December 2020 (Baker et al., 2021; Senedd Research, 2022; SPICe Spotlight, 2022). The final full lockdown started in Wales and Northern Ireland in December 2020, joined by Scotland and England in January 2021 and ended in March 2021 (Baker et al., 2021; Senedd Research, 2022; SPICe Spotlight, 2022). While the specific restrictions and timing of lockdowns varied between the UK nations of England, Scotland, Wales, and Northern Ireland, the main lockdown restrictions were similar throughout the UK, including rules such as reductions in gatherings, movement, and limited services (Ferguson et al., 2021). Legal restrictions on gatherings, travel and enforced social isolation continued throughout 2021 with all restrictions finally lifting in January 2022 for Scotland (Scotland to lift most remaining COVID‐19 restrictions, 2022); February 2022 for England and Northern Ireland (COVID‐19: Remaining restrictions in NI are lifted, 2022; UK Health Security Agency, 2022), and Wales in May 2022 (Culbertson, 2022).

### Child behaviours that challenge

During COVID‐19 lockdown, subgroups of families already disadvantaged by health inequalities were at higher risk of poorer mental health outcomes than others. One of these disadvantaged family groups included families who have children with intellectual disability (Diaz et al., 2021). Intellectual disability is characterised by social, cognitive, and adaptive skill difficulties (Zayac & Johnston, 2008), manifesting as self‐injury, sexually or socially inappropriate behaviours, physical and/or verbal aggression, disturbed sleep, over or in activity, and destructive tendencies (Benson & Brooks, 2008; Myrbakk & Von Tetzchner, 2008). These behaviours have also been referred to in the literature as ‘challenging behaviours’ or ‘behaviours that challenge’ (Myrbakk & Von Tetzchner, 2008). The National Institute for Health and Care Excellence (NICE, 2015) highlights that behaviours that challenge do not constitute a diagnosis, and may serve an important purpose to the individual with an intellectual disability (e.g., communication or sensory stimulation). Despite this, some of these behaviours (specifically self‐injury and aggressive outbursts) can present persistent difficulties for family, carers, practitioners, and services. In addition, the health inequalities these children face pre‐date COVID‐19 and include, poorer general health, psychiatric disorders, and higher rates of emotional and behavioural difficulties compared to their typically developing peers (Allerton et al., 2011; Emerson & Einfeld, 2010). Disruption to routine and reduction in access to enjoyed activities (situations such as those observed during COVID‐19 lockdown) are known to further exacerbate behaviours that challenge, which may also cause the development of other mental health difficulties including psychological distress (Borthwick‐Duffy, 1994; NICE guidelines—NG11; published May, 2015).

### Caregiver psychological distress

Parenthood and caregiving are often characterised by constant change that is driven by the developmental stage of the child (O’Connor, 2002) indicating that caregivers must always be adaptive to the child’s needs. In addition, parents and caregivers experience more negative responses to disasters compared to those with fewer caregiving responsibilities (e.g. Fussell & Lowe, 2014) indicating that caregiving groups are at heightened risk of poorer outcomes during times of adversity. Caregivers of children who have intellectual disability specifically, are a vulnerable group who are more likely to develop mental health difficulties compared to carers of typically developing children (Gallagher et al., 2008; Herring et al., 2006). Social support and the severity of the disability, psychiatric disorder and child’s levels of challenging behaviours are all key factors that contribute to caregiver psychological distress (e.g., Blacher & McIntyre, 2006; Unwin & Deb, 2011; Weiss, 2002; White & Hastings, 2004). Management of challenging behaviours impacts both the individuals’ quality of life and opportunities (e.g., social, education, career) but also poses a significant additional burden on caregivers, leading to increased stress levels (Blacher & McIntyre, 2006; McConnell & Savage, 2015). Perceived burden includes guilt, resentment, embarrassment, isolation, and loss of control (Zarit et al., 1980), with guilt being the strongest predictor of anxiety and depression in caregivers of children with intellectual disability (Gallagher et al., 2008). COVID‐19 has been reported to have significantly increased the perceived levels of strain and burden in caregivers who have children with Special Educational Needs and Disabilities (SEND) (Dhiman et al., 2020).

Respite services and social support are crucial for reducing caregiver stress and burnout (Dunn et al., 2001; Sherman, 1995), with research indicating that caregivers who have high levels of social support show better psychological adjustment (Duis et al., 1997; Dunn et al., 2001). Contrastingly, greater distress is reported in families who perceive high caregiver burden and limited social support (Dunn et al., 2001; Gallagher et al., 2008). Therefore, significant reductions in educational, clinical and respite services for children with intellectual disabilities during COVID‐19 presented significant challenges to caregivers (Neece et al., 2020; O’Hagan & Kingdom, 2020) and some of these reductions have continued despite the easing of lockdown measures (Gillespie‐Smith et al., 2021).

#### Caregiver coping

Previous research has suggested that disruption to routines and restrictions impact how caregivers perceive their situation and influences the coping strategies they employ (Cramm & Nieboer, 2011). According to general stress and coping theory (e.g., Lazarus & Folkman, 1984) parents of children who have intellectual disability adapt to external situations using individual coping strategies (e.g., Hastings et al., 2005). Coping is context dependent (Carver et al., 1989); therefore, interaction between stress and the contextual environment will affect the development of coping strategies (Hastings et al., 2005). To date, research has reported coping strategies that positively impact mental health outcomes in carers of children with intellectual disability, include active emotional coping (Ganjiwale et al., 2016); cognitive reframing and acceptance (Hastings et al., 2002; O’Ó Donnchadha, 2018); and positive re‐interpretation (Smith, et al., 2008). In the literature three ways of coping are often presented: (a) problem focussed coping, which refers to actively eliminate sources of stress and manage stressful events; (b) emotion focussed coping, which includes efforts to reduce the emotional consequences of stressful situations; (c) avoidant coping, which indicates physical or cognitive efforts to disengage from the stressful event or situation (Dias et al., 2012; Folkman, 2008). Research to date has shown that problem‐based coping strategies are related to good mental health, while emotion focussed or avoidant coping strategies are correlated with poor mental health (Lloyd & Hastings, 2008; Macdonald, 2011; Smith et al., 2008). The way in which caregivers deal with stressful events can ultimately present a risk for, or protect them against, poor mental health outcomes.

In addition, caregiver distress is significantly associated with an increase in behavioural and emotional problems in children with intellectual difficulties (Bailey et al., 2021; Totsika et al., 2020). To our knowledge, only one study to date has looked at both child challenging behaviours and caregiver distress in the same UK based sample during COVID‐19 (Gillespie‐Smith et al., 2021). They found that parents of children with intellectual disability who utilised high levels of avoidant coping strategies, such as denial and behaviour disengagement, went on to show higher levels of psychological distress. As this was a cross‐sectional study, reports of child and caregiver distress are limited to only one time‐point during the COVID‐19 lockdown.

### Current study

The current study aimed to extend the research carried out by Gillespie‐Smith et al. (2021) by exploring longitudinally whether caregiver coping strategies continued to relate to mental health in caregivers of children with intellectual disability during and after the COVID‐19 UK lockdown. The current study looks more specifically at how coping strategies associated with types of caregiver psychological distress (stress, anxiety, and depression). The following hypotheses were posed:

1. That socio‐demographic factors such as caregiver education level and employment status would be related to caregiver coping, stress, anxiety and depression.
2. Due to the unprecedented nature of the pandemic, and lack of control over events, it was predicted that higher levels of avoidant, problem focussed and emotion focussed coping, would be observed during lockdown (T1) compared to after lockdown (T2) and that all coping strategies would be higher for caregivers who have children with Intellectual disabilities compared to caregivers who have children who are typically developing.
3. It was also predicted that caregiver distress (stress, anxiety and depression) would be significantly higher during lockdown (T1) compared to after lockdown (T2), and that all domains of distress would be higher for caregivers who have children with Intellectual disabilities compared to caregivers who have children who are typically developing.
4. It was predicted that child challenging behaviours would be significantly higher during lockdown (T1) compared to post‐lockdown (T2) and that behaviours that challenge would be reported to be higher in the caregiver group who have children with Intellectual disabilities compared to the caregiver group who have children who are typically developing.
5. It was further predicted that child challenging behaviours would be related to caregiver stress, anxiety and depression as well as caregiver coping strategies (avoidant, problem focussed and emotion focussed) during T1 and T2.
6. Finally, it was predicted that higher levels of avoidant and emotion focussed coping and lower levels of problem focussed coping would predict higher levels of stress, anxiety and depression in caregivers during both time periods.

## METHODS

### Participants

Participants were parents or caregivers (hereafter referred to as ‘caregivers’) of a child with or without an intellectual disability between 5 and 19 years old. Both caregivers of neurotypical children and children who have intellectual disabilities were recruited through research adverts posted on social media platforms (Facebook and Twitter). Caregivers of children with intellectual disabilities were additionally recruited through UK based charity (e.g. Down Syndrome Foundation, ENABLE, Scottish Autism, National Autistic Society, Williams Syndrome Foundation) and parent groups (e.g. Kindred, Parent and Carer Alliance) by sharing the study advert via charity websites, newsletters or mailing lists. To be included in the current sample, caregivers were required to be 16 years and above, understand the English language, and live in the UK. To reduce the risk of bot or false responses, participants had to first contact the researcher and tell them where they had seen the study advert, provide their child’s birthday, whether they had disability and if so provide information about their disability. Participants were then sent a link to take part in the survey during Time Period 1 (T1; August‐December 2021) and the same caregivers were invited via email to take part in the survey again during Time Period 2 (T2; January–March 2022). No participants declined to take part in T1 however 62 participants did not complete the follow up T2 survey (despite 3 reminder emails being sent). Only participants that took part in T1 were invited to take part in T2. 182 participants took part in T1 survey initially, however during data inspection, *n* = 11 responses were judged to be invalid (64% of these were completed in less than 900 s, and 36% were responses from a matching IP address). The final sample included 171 participants in T1 and 109 participants in T2. Since 36.3% of participants did not complete the T2 study, missing data analysis was carried out to check whether values were missing completely at random (MCAR). Little’s MCAR test (Little, 1988) was not significant, Chi‐Square (171, 5) = 3.57, *p* = 0.613, suggesting that values were missing entirely at chance. Furthermore, independent samples *t*‐tests were further carried out to check whether the participants who did not take part in T2 significantly differed from those who did take part in T2 based on the key variables, parental distress, coping strategies and levels of child behaviours that challenge. There were no significant differences. See Table 1 for sociodemographic characteristics of the caregivers and their children for the intellectual disability and the group reporting no intellectual disabilities during the two time points (hereafter referred to as ‘typically developing’). Despite the caregiver groups being randomly recruited, the final sample sizes numbers were relatively similar.

**TABLE 1:** Sociodemographic characteristics as a percentage of the groups with versus without Intellectual Disabilities (ID), and variable means at Time Period 1 and Time Period 2.

	T1		T2	
	With ID (*n* = 90)	Without ID (*n* = 81)	With ID (*n* = 58)	Without ID (*n* = 51)
Characteristic	N (%)	N (%)	N (%)	N (%)
Child’s age				
5–7	19 (21.1)	16 (19.8)	4 (6.9)	13 (25.5)
8–10	25 (27.8)	18 (22.2)	17 (29.3)	11 (21.6)
11–13	13 (14.4)	16 (19.8)	17 (29.3)	10 (19.6)
14–16	20 (22.2)	16 (19.8)	8 (13.8)	10 (19.6)
17–19	6 (6.7)	6 (7.4)	11 (19.0)	6 (11.8)
Did not disclose	7 (7.8)	9 (11.1)	1 (1.7)	0 (0.0)
Child’s gender				
Male	60 (66.7)	45 (55.6)	37 (63.8)	29 (56.9)
Female	29 (32.2)	35 (43.2)	21 (36.2)	21 (41.2)
Non‐binary	1 (1.1)	1 (1.2)	0 (0.0)	1 (2.0)
Number of siblings				
0	23	19 (23.5)	12 (20.7)	10 (19.6)
1	35	39 (48.1)	23 (39.7)	24 (47.1)
2	20	19 (23.5)	15 (25.9)	15 (29.4)
3	7	2 (2.5)	4 (6.9)	2 (3.9)
4	2	2 (2.5)	1 (1.7)	0 (0.0)
5+	2	0 (0.0)	2 (3.4)	0 (0.0)
Did not disclose	1	0 (0.0)	1 (1.7)	0 (0.0)
Caregiver’s educational level				
No formal qualification	2	0 (0.0)	1 (1.7)	0 (0.0)
School leaving certificate (e.g GCSE or A levels, highers, high school diploma or certificate)	10	13 (16.0)	6 (10.3)	6 (11.8)
Further vocational training for a specific industry	18	8 (9.9)	14 (24.1)	6 (11.8)
University degree or equivalent (e.g. BSc, BA, etc.)	37	34 (42.0)	26 (44.8)	24 (47.1)
Postgraduate university degree or equivalent (e.g. MA, MSc, PhD, DClin, etc.)	20	23 (28.4)	9 (15.5)	14 (27.5)
Other	3	2 (2.5)	2 (3.5)	1 (2.0)
Did not disclose	0	1 (1.2)	0 (0.0)	0 (0.0)
Caregiver’s employment				
Homemaker	19	12 (14.8)	14 (24.1)	8 (15.7)
Volunteer work	2	0 (0.0)	1 (1.7)	0 (0.0)
Part time paid employment	32	28 (34.6)	20 (34.5)	18 (35.3)
Full time paid employment	22	36 (44.4)	15 (25.9)	21 (41.2)
Unemployed	2	0 (0.0)	1 (1.7)	0 (0.0)
Student	3	1 (1.2)	1 (1.7)	1 (2.0)
Other	10	4 (4.9)	6 (10.3)	3 (5.9)
Variable means (*sd*)				
Child behaviours that challenge	6.661 (0.708)	5.795 (0.710)	4.391 (1.075)	3.073 (1.269)
Problem focussed coping	4.383 (0.558)	4.420 (0.562)	4.165 (0.578)	4.071 (0.691)
Avoidant coping	3.799 (0.442)	3.638 (0.533)	3.757 (0.484)	3.649 (0.444)
Emotion focussed coping	5.053 (0.546)	5.011 (0.587)	5.084 (0.557)	5.112 (0.519)
Caregiver stress	4.215 (0.659)	3.842 (0.604)	3.053 (1.136)	2.676 (1.037)
Caregiver anxiety	3.654 (0.673)	3.258 (0.575)	2.231 (1.258)	1.763 (1.237)
Caregiver depression	3.839 (0.706)	3.509 (0.633)	2.612 (1.179)	2.189 (1.154)

*Note*: Table shows the demographic percentages of the 2 groups (caregivers of neurotypical children and caregivers of children with Intellectual Disability) during Time Period 1 (T1; August‐December 2021, *n* = 171); Time Period 2 (T2; January‐March 2022, *n* = 109) and the variable means at Time Period 1 and Time Period 2.

A description of the intellectual disabilities group diagnoses for T1 can be found in Table 2. A G‐power calculation based on regression analyses with 8 predictors (controlling for variables gender and age of child), with a moderate effect size of 0.3 indicated that a sample of *n* = 107 was required. The final sample sizes at both time points remained within these parameters.

**TABLE 2:** Diagnosis as a percentage of the group with Intellectual Disability at T1 and T2.

	T1 *n* = 90		T2 *n* = 58	
Diagnosis	Number	Percentage	Number	Percentage
Autism	28	31.1	17	29.3
Autism and ADHD or developmental delay	10	11.1	9	15.5
ADHD	3	3.3	3	5.2
Down syndrome	11	12.2	8	13.8
Williams syndrome	11	12.2	8	13.8
Intellectual disability	10	11.1	5	8.6
Global/Developmental delay	4	4.4	3	5.2
Williams syndrome and autism	1	1.1	1	1.7
Rare chromosome Abnormality	2	2.2	2	3.4
Fragile X syndrome	2	2.2	1	1.7
Coffin siris syndrome and autism	1	1.1	‐	‐
Down syndrome and autism	2	2.2	1	1.7
Rubinstein‐taybi syndrome and autism	1	1.1	‐	‐
Cornelia de lange syndrome	4	4.4	‐	‐

*Note*: Table shows the diagnosis percentages of the group with Intellectual Disability during Time Period 1 (T1; August‐December 2021, *n* = 171) and then again during Time Period 2 (T2; January‐March 2022, *n* = 109).

### Procedure

Following ethical approval granted from the University of XXXX Ethics Committee, participants were recruited online (via online social networking sites, charity websites, and parent group pages). To take part parents had to contact the researcher who then sent them a direct link to the online survey hosted by Qualtrics survey system which took approximately 45–50 min to complete. After clicking a link, participants were first presented with the participant information screen, followed by the consent screen before entering the survey battery. Due to the sensitive nature of the survey mental health support information was provided throughout the survey including the participant information screen, and again at the end within the debrief screen. Participants were also informed that they were free to exit at any point (with no negative repercussions) by closing the computer window.

### Measures

#### Survey on COVID‐19

A questionnaire was developed, comprising 40 demographic data items (caregiver age, child age, marital status, employment status, number of siblings etc.) in addition to categorical questions on the impact of COVID‐19 and how the government restrictions had impacted their family situation, living arrangements, and services/respite support. The survey took approximately 10 min to complete and was completed in T1 data collection only. Following this, data were transformed to numerical form, by applying scoring schemes to the nominal data so these could be inputted to parametric tests. This T1 data was linked to the T2 data using email addresses. Caregivers were then asked to complete the following psychometric scales during the two time periods.

#### Caregiver psychological distress

The Depression, Anxiety and Stress Scale (DASS‐21; Lovibond & Lovibond, 1995), a self‐report questionnaire consisting of 21 items, with 7 items per subscale: stress, anxiety and depression. Participants are asked to score every item on a three‐point Likert scale from 0 (did not apply to me at all) to 3 (applied to me very much), and scores from each item were added to obtain a total for each subscale. Numerous studies have reported favourable psychometric properties of the DASS in adults with anxiety and/or mood disorders in both community and clinical samples (Antony et al., 1998; Clara, et al., 2001). DASS subscale scores were used as a measure of stress, anxiety and depression with higher scores indicating increased severity (Beaufort, et al., 2017). Studies have demonstrated excellent internal consistency of the DASS‐21 items, ranging from 0.81 to 0.97 (Gloster, et al., 2008). In the current study, the DASS‐21 displayed robust internal consistency at Time 1 (*a* = 0.91) and Time 2 (*a* = 0.94).

#### Child behaviours that challenge

The Developmental Behaviour Checklist—P24 (DBC—P24; Taffe, et al., 2007) is a 24‐item checklist which is a shorter form of the DBC which is a 96‐item scale (Einfeld & Tonge, 1995, 1995, 1995) specifically designed to assess behavioural and emotional disturbance in children/adolescents with intellectual disability. The instrument has 24 items (e.g., over‐excitement, impatience, and lack of affection) that are scored based on a three‐point Likert scale from 0 (*not true*) to 2 (*very true*). The DBC‐P24 performs very well in terms of low bias and high precision in cross‐validation and displays excellent sensitivity and specificity properties (Taffe et al., 2007). A total Child Challenging Behaviour score was calculated by summing the scores with higher scores indicating higher levels of child ‘challenging behaviours’. In the current study, the DBC‐P24 displayed robust internal consistency at Time 1 (*a* = 0.91) and Time 2 (*a* = 0.91).

#### Caregiver coping strategies

The Brief COPE (Carver, 1997) a shortened version of the COPE, asks 28 questions on a 4‐point Likert scale, ranging from 1 (*I haven’t been doing this at all)* to 4 (*I’ve been doing this a lot*), where 2 items each form 14 sub‐scales with the totals of each subscale indicating how often the coping strategy is used. Each subscale has shown good internal consistency indicated by the Cronbach’s alpha values ranging from 0.50 to 0.90 (Carver, 1997). In the current study, the Brief COPE displayed robust internal consistency at Time 1 (*a* = 0.84) and Time 2 (*a* = 0.85). The subscales include active coping, planning, positive reframing, acceptance, humour, turning to religion, using emotional support, using instrumental support, self‐distraction, denial, venting, substance use, behavioural disengagement, and self‐blame. This scale has been used widely in groups of caregivers of children with intellectual disability and developmental disorders (e.g. Ganjiwale et al., 2016; Isa et al., 2017; Panicker and Ramesh, 2019). The scale provided information on each caregiver’s abilities to cope, perceived social support and self‐regulation. Higher scores reflect a higher tendency to implement the specified coping strategies.

These coping strategies were divided up to form three key coping strategies based on the three‐factor model of Brief COPE (Dias et al., 2012) which has also shown good validity (Poulus et al., 2020). These three factors include (a) problem focussed coping, (b) emotion focussed coping, and (c) avoidant coping. Problem focussed coping consists of active coping, instrumental support, planning and positive reframing. These coping strategies are aimed at changing the stressful situation and high scores are indicative of psychological strength. Emotion focussed coping is characterised by venting, use of emotional support, humour, acceptance, self‐blame, and religion. Higher scores indicate coping strategies that aim to regulate emotions associated with the stressful event. Finally, avoidant coping is characterised by the facets of self‐distraction, denial, substance use, and behavioural disengagement. Higher scores on this coping strategy indicate physical or cognitive efforts to disengage from the stressful event or situation. Lower scores within this category are therefore indicative of adaptive coping.

### Data analysis

Normality was assessed using the Shapiro‐Wilk test which showed that for both time points (Time 1 and Time 2) child behaviours that challenge, avoidant coping, caregiver stress, anxiety and depression significantly departed from normality (ps < 0.05). List‐wise deletion was used for missing data. Visual inspections of histograms and Q‐plots showed that the data were positively skewed, however, the skewness was still within typical parameters (all less than 1). Square root transformations were still applied before Pearson Correlations were carried out separately for each group (caregivers of children with intellectual disability and typically developing children). Correlations were carried out to highlight significant relationships between demographics, coping strategies and caregiver psychological distress. Bonferroni corrections for multiple comparisons were also applied throughout. Mixed 2 × 2 × 3 ANOVAs were carried out with between subject variable being group (caregivers of children who have ID vs. caregivers of typically developing children) and within subject variable being time (T1 and T2) and caregiving coping (avoidant vs. emotion focussed vs. problem focussed). Similarly, mixed 2 × 2 × 3 ANOVAs were carried out with between subject variable being group (caregivers of children who have ID vs. caregivers of typically developing children) and within subject variable being timepoint (T1 and T2) and caregiver mental health outcome (stress, anxiety and depression). A mixed 2 × 2 ANOVA was also carried out with between subject factor being group (caregivers of children who have ID vs. caregivers of typically developing children) and within subject being timepoints (T1 and T2) with dependent variable being child behaviours that challenge. Significant interactions were further explored with post‐hoc independent samples *t*‐tests. To understand whether coping strategies associated with poor caregiver mental health, Pearson’s correlations were carried out (with Bonferroni corrections applied for multiple comparisons) highlighting coping variables that had significant relationships with caregiver stress, anxiety and depression. Significant variables were taken forward into multiple linear regressions analyses. A series of six hierarchical regression analyses were conducted, to determine the independent effect of group (ID or TD), level of child behaviours that challenge and specific coping strategies on caregiver stress, anxiety and depression. In each model, group was entered as a dichotomous variable at step 1, child behaviour (DBC scores) at step 2 and coping strategy at step 3. The two significant coping strategies (avoidant and emotion focussed) were then entered for stress, anxiety and depression outcome variables. Multiple analyses were conducted to avoid inter‐collinearity with coping strategies and caregiver distress domains.

## RESULTS

### Descriptive statistics

#### Demographic data

There were no significant correlations between demographic data, child behaviours that challenge, caregiver coping and caregiver psychological distress (see Table 3).

**TABLE 3:** Pearson’s correlations between caregiver demographics, coping strategies and psychological distress at Time period 1 (T1) and Time period 2 (T2).

	2. No. of siblings	3. Highest parent qualification	4. Parent marital status	5. Employment	6. T1 COPE problem focused	7. T1 COPE avoidant	8. T1 COPE emotion focussed	9. T1 stress	10. T1 anxiety	11. T1 depression	12. T2 COPE problem focussed	13. T2 COPE avoidant	14. T2 COPE emotion focussed	15. T2 stress	16. T2 anxiety	17. T2 depression
1. Age in months	0.056	−0.036	−0.066	−0.020	0.006	−0.139	−0.083	−0.038	0.010	−0.034	−0.046	−0.151	−0.044	−0.100	0.036	−0.159
2. No. of siblings	_	−0.191	0.000	0.004	−0.030	0.043	0.007	0.150	0.131	0.209*	0.059	0.102	0.014	0.184	0.207	0.211
3. Highest parent qualification		_	0.101	0.099	−0.017	−0.036	−0.036	−0.049	−0.131	−0.092	0.000	−0.060	−0.186	−0.186	−0.251*	−0.133
4. Parent marital status			_	−0.020	0.138	−0.054	0.017	0.026	−0.036	−0.016	0.103	0.163	0.113	−0.089	0.004	−0.019
5. Employment				_	0.068	−0.070	−0.008	−0.049	−0.008	−0.078	−0.030	0.007	−0.013	−0.115	−0.052	−0.077

*Note*: Pearsons correlations between demographics (including age of child, no. of siblings, highest parental qualification, parental marital status and employment) with Time period 1 (T1) and Time period 2 (T2) caregiver coping strategies (COPE problem focussed; avoidant; emotion focussed) and caregiver psychological distress (stress, anxiety and depression).

*. Correlation is significant to 0.01 **. Correlation is significant to 0.003 (following Bonferroni Correction; two tailed).

### Caregiver coping between groups and between timepoints

Mixed ANOVA showed that there was a significant effect of time F (1, 107) = 5.391, *p* = 0.022, *Np* ^2^ = 0.048, SE = 0.427, indicating that there were higher levels of coping strategies during T1 (*m* = 4.374, SE = 0.050) compared to T2 (*m* = 4.153, SE = 0.060). There was no significant time × group interaction F (1, 107) = 0.002, *p* = 0.967, *Np* ^2^ = 0.000, SE = 0.051). There was a significant effect of coping F (2, 192) = 402.027, *p* < 0.001, *Np* ^2^ = 0.790, SE = 0.529. Paired samples *t*‐tests revealed that caregivers engaged in avoidant coping (*m* = 3.736, SE = 0.036 significantly less compared to problem focussed coping (*m* = 4.052, SE = 0.050) *t* (170) = 5.283, *p* < 0.001, *d* = 0.782., and emotion focussed coping (*m* = 5.039, SE = 0.040), t (170) = 32.388, *p* < 0.001, *d* = 0.526. Emotion focussed coping was the strategy most used by caregivers, with them engaging in this strategy significantly more than problem focussed coping *t* (170) = 21.507, *p* < 0.001, *d* = 0.601. There was no significant interaction between group and coping showing that both groups employed similar coping strategies F (2, 192) = 1.003, *p* = 0.361, *Np* ^2^ = 0.009. There was a significant interaction between time and coping F (2, 189) = 20.082, *p* < 0.001, *Np* ^2^ = 0.158. To explore this significant interaction paired samples *t*‐tests were carried out which showed that the only significant difference was problem focussed coping which was significantly higher in T1 (*m* = 4.365, SE = 0.047) compared to T2 (*m* = 3.654, SE = 0.084), *t* (143) = 8.626, *p* < 0.001, *d* = 0.990. There was no significant interaction between timepoint, coping and group F (2, 189) = 1.848, *p* = 0.165, *Np* ^2^ = 0.017. There was no overall significant effect of group F (1, 108) = 0.575, *p* = 0.450, *Np* ^2^ = 0.005.

### Caregiver psychological distress between groups and timepoints

Mixed ANOVA showed that there was a significant effect of timepoint F (1, 107) = 247.196, *p* < 0.001, *Np* ^2^ = 0.698, SE = 1.154, indicating that there were higher levels of psychological distress during T1 (*m* = 3.731, SE = 0.064) compared to T2 (*m* = 2.434, SE = 0.113). There was no significant time × group interaction, F (1, 107) = 0.118, *p* = 0.732, *Np* ^2^ = 0.001. There was a significant effect of psychological distress F (2, 201) = *p* < 0.001, *Np* ^2^ = 0.401. Paired samples *t*‐tests were carried out to explore this significant effect and it showed that caregivers experienced lower levels of anxiety (*m* = 3.023, SE = 0.069) compared to stress (*m* = 3.299, SE = 0.058) *t* (170) = 9.063, *p* < 0.001, *d* = 0.397 and depression (*m* = 3.320, SE = 0.068) *t* (170) = 6.103, *p* < 0.001, *d* = 0.635. There was no significant interaction between group and caregiver distress F (2, 201) = 0.146, *p* = 0.852, *Np* ^2^ = 0.001. There was a significant interaction between timepoint and distress F (2, 208) = 8.034, *p* < 0.001, *Np* ^2^ = 0.070. Paired samples *t*‐tests were carried out to explore this significant interaction. Stress was found to be significantly higher in T1 (*m* = 4.040, SE = 0.063) compared to T2 (*m* = 2.876, SE = 0.106), *t* (108) = 13.325, *p* < 0.001, *d* = 0.912; Anxiety was also significantly higher in T1 (*m* = 3.469, SE = 0.063) compared to T2 (*m* = 2.012, SE = 0.121) *t* (108) = 15.329, *p* < 0.001, *d* = 0.992; and depression was significantly higher during T1 (*m* = 3.684 SE = 0.066) compared to T2 (*m* = 2.414, SE = 0.113) *t* (108) = 13.372, *p* < 0.001, *d* = 0.992. There was no significant interaction between timepoint, distress and group F (2, 208) = 0.200, *p* = 0.813, *Np* ^2^ = 0.002. There was an overall significant effect of group F (1, 107) = 8.004, *p* = 0.006, *Np* ^2^ = 0.070, indicating that the group of caregivers who have children with ID showed higher distress (*m* = 3.337, SE = 0.079) compared to caregivers who have typically developing children (*m* = 3.077, SE = 0.091).

### Child challenging behaviours between groups and between timepoints

The ANOVA showed that there was a significant effect of timepoint F (1, 107) = 1208.532, *p* < 0.001, *Np* ^2^ = 0.919 indicating that the children showed higher levels of behaviours that challenge during T1 (*m* = 6.256, SE = 0.079) compared to T2 (*m* = 3.774, SE = 0.128). There was a significant interaction between group and timepoint F (1, 107) = 9.919, *p* = 0.002, *Np* ^2^ = 0.085. Independent samples *t*‐tests highlighted that the caregivers of children with intellectual disabilities reported significantly higher levels of child challenging behaviours during T1 (*m* = 6.661, SE = 0.093) compared to T2 (*m* = 4.391, SE = 0.141) *t* (57) = 26.742, *p* < 0.001, *d* = 0.647. There was a significant difference in levels of challenging behaviours in the caregivers who had typically developing children during T1 (*m* = 5.795, SE = 0.099) compare to T2 (*m* = 3.073, SE = 0.177), *t* (50) = 22.892, *p* < 0.001, *d* = 0.849. There was also an overall effect of Group F (1,107) = 40.692, *p* < 0.001, *Np* ^2^ = 0.276, indicating that caregivers of children with intellectual disabilities (*m* = 5.526, SE = 0.171) reported significantly higher level of challenging behaviours compared to caregivers of children who are typically developing (*m* = 4.434, SE = 0.190).

### Correlations

#### Time one

**Intellectual disabilities group:** Child challenging behaviours were positively associated with the following caregiver measures: stress (*r* = 0.441, *p* < 0.001, SE = 0.086), anxiety (*r* = 0.519, *p* < 0.001, SE = 0.078) depression (*r* = 0.446, *p* < 0.001, SE = 0.086), avoidant coping (*r* = 0.477, *p* < 0.001, SE = 0.083) and emotion focussed coping (*r* = 0.341, *p* <. 001, SE = 0.013). Similarly, caregiver stress levels were associated with avoidant coping (*r* = 0.449, *p* < 0.001, SE = 0.086), emotion focussed coping (*r* = 0.372, *p* < 0.001, SE = 0.092). Caregiver anxiety level was associated with avoidant coping (*r* = 0.573, *p* < 0.001, SE = 0.072), and emotion focussed coping (*r* = 0.390, *p* < 0.001, SE = 0.091). Depression was associated with avoidant coping only (*r* = 0.526, *p* < 0.001, SE = 0.078).

**Typically developing group:** Child challenging behaviours were positively associated with caregiver stress (*r* = 0.323, *p* = 0.003, SE = 0.102), anxiety (*r* = 0.427, *p* < 0.001, SE = 0.093) and depression (*r* = 0.409, *p* < 0.001, SE = 0.094). There was also a positive association between child behaviours that challenge and caregiver avoidant coping (*r* = 0.470, *p* < 0.001, SE = 0.088). Caregiver stress was also positively associated with avoidant coping (*r* = 0.538, *p* < 0.001, SE = 0.081) and emotion focussed coping (*r* = 0.372, *p* < 0.001, SE = 0.098). Caregiver anxiety and depression were related to avoidant coping only (*r* = 0.542, *p* < 0.001, SE = 0.080; *r* = 0.591, *p* < 0.001, SE = 0.074 respectively).

#### Time two

**Intellectual disabilities group:** There was a significant positive association between child challenging behaviours and caregiver anxiety (*r* = 0.496, *p* < 0.001, SE = 0.102); as well as caregiver avoidant coping strategies (*r* = 0.377, *p* = 0.004, SE = 0.116). Caregiver distress was also related to several coping strategies; caregiver stress was positively associated with avoidant coping (*r* = 0.382, *p* = 0.003, SE = 0.115) and emotion focussed coping (*r* = 0.380, *p* = 0.003, SE = 0.115). Caregiver anxiety was related to avoidant coping (*r* = 0.373, *p* = 0.004, SE = 0.116); and emotion focussed coping (*r* = 0.371, *p* = 0.004, SE = 0.116). Depression was related to avoidant coping only (*r* = 0.470, *p* = 0.004, SE = 0.105).

**Typically developing group:** In the typically developing group child challenging behaviours were positively associated with caregiver anxiety (*r* = .386, *p* = .005, SE = .123), and caregiver avoidant coping (*r* = .460, *p* < .001, SE = .114) and emotion focused coping (*r* = .464, *p* < .001, SE = .113). In addition, caregiver stress (*r* = .405, *p* < .001, SE = .121), Anxiety (*r =* .460, *p* < .001, SE = .114); and depression (*r* = .568, *p* < .001, SE = .098) were all positively associated with caregiver avoidant coping only.

### Regression analyses

#### Time 1

##### Caregiver stress

Table 4 displays the results of the hierarchical regression where group was added at step 1, child behaviour at step two and avoidant coping at step 3. The first model with group being added was significant, *F* (1, 169) = 7.440, *p* = 0.007, SE = 0.103. The addition of child behaviour in model 2 explained an additional 14% of variance in caregiver stress, after controlling for group, Δ *R* ^2^ = 0.14, *F* change = 27.822 (2, 168), *p* < 0.001, SE = 0.066. In the final model, avoidant coping was statistically significant, *F* change = 29.441 (1, 167), *p* < 0.001, SE = 0.099. The final model explains 30% of the variance in caregiver stress *R* ^2^ = 0.30. Table 2 also shows the same analysis with emotion focussed coping replacing avoidant coping. Results of the next significant model with emotion focussed coping being added at Step 3, *F* (1, 167) = 16.503, *p* < 0.001, SE = 0.009. This model explains 25% of the variance (*R* ^2^ = 0.25).

**TABLE 4:** Hierarchical regression summary: Caregiver stress, anxiety and depression at time 1.

			95% CI					
Outcome	Variable	B	LL	UL	SE B	β	*R* ^2^	(Δ) *R* ^2^
Caregiver	*Step1*							
Stress	Constant	3.59	3.26	3.92	0.17			
	Group	0.28	0.08	0.49	0.10	0.21	0.04	0.04
	*Step 2*							
	Constant	1.80	1.07	2.54	0.37			
	Group	0.01	−2.09	0.22	0.11	0.01		
	DBC	0.35	0.22	0.48	0.07	0.42	0.18	0.14
	*Step 3 for AC*							
	Constant	0.77	−0.00	1.55	0.39			
	Group	0.10	−0.10	0.30	0.10	0.73		
	DBC	0.17	0.03	0.31	0.07	0.21		
	AC	0.54	0.34	0.73	0.10	0.40	0.30	0.12
	*Step 3 for EFC*							
	Constant	1.34	0.60	2.08	0.37			
	Group	0.03	−0.18	0.24	0.11	0.02		
	DBC	0.28	0.14	0.41	0.07	0.33		
	EFC	0.04	0.02	0.05	0.01	0.04	0.25	0.07
Caregiver	*Step1*							
Anxiety	Constant	3.08	2.76	3.41	0.16			
	Group	0.26	0.06	0.47	0.10	0.20	0.04	0.04
	*Step 2*							
	Constant	0.88	0.18	1.57	0.35			
	Group	−0.08	−0.28	0.13	0.10	−0.06		
	DBC	0.43	0.31	0.56	0.06	0.52	0.25	0.21
	*Step 3 for AC*							
	Constant	−0.20	−0.92	0.52	0.36			
	Group	0.02	−0.16	0.21	0.10	0.17		
	DBC	0.24	0.12	0.37	0.06	0.30		
	AC	0.56	0.38	0.75	0.09	0.42	0.39	0.14
	*Step 3 for EFC*							
	Constant	0.64	−0.84	1.36	0.37			
	Group	−0.06	−0.27	0.14	0.10	−0.05		
	DBC	0.39	0.27	0.52	0.07	0.48		
	EFC	0.02	0.01	0.04	0.01	0.15	0.27	0.02
Caregiver	*Step1*							
Depression	Constant	3.37	3.03	3.72	0.18			
	Group	0.23	0.02	0.45	0.11	0.16	0.03	0.03
	*Step 2*							
	Constant	1.24	0.48	2.00	0.39			
	Group	−0.10	−0.32	0.13	0.11	−0.07		
	DBC	0.42	0.28	0.55	0.07	0.48	0.20	0.18
	*Step 3*							
	Constant	0.01	−0.77	0.79	0.39			
	Group	0.02	−0.19	0.22	0.10	0.01		
	DBC	0.20	0.07	0.34	0.07	0.23		
	AC	0.64	0.45	0.84	0.10	0.46	0.36	0.16

*Note*: Hierarchical model was carried out, group was entered as a dichotomous variable at step 1, child behaviours that challenge (DBC scores) at step 2 and coping strategy—avoidant coping at step 3; or Emotion Focussed coping at step 3 with caregiver stress, anxiety and depression as the outcomes.

Abbreviations: AC, avoidant coping subscale; CI, Confidence interval; DBC, Developmental Behaviour Checklist; EFC, emotion focussed coping subscale; IL, upper limit; LL, lower limit.

##### Caregiver anxiety

Table 4 also displays the results of the hierarchical regression where group was added at step 1, child behaviour at step two and avoidant coping at step 3. The first model with group as the independent variable, was significant, *F* (1, 169) = 6.658, *p* = 0.011, SE = 0.102. The addition of child behaviour in model 2 explained an additional 21% of variance in caregiver anxiety, after controlling for group, Δ *R* ^2^ = 0.21, *F* change = 47.354 (2, 168), *P* < 0.001, SE = 0.063. In the final model, avoidant coping was statistically significant, F change = 37.718 (1, 167). *P* < 0.001. SE = 0.092. The final model explains 39% of the variance in caregiver anxiety *R* ^2^ = 0.39. Another significant model emerged when emotion focussed coping was added at Step 3 F (1, 167) = 4.607, *p* = 0.033, explaining 27% of the variance (*R* ^2^ = 0.27)—see Table 4.

##### Caregiver depression

Table 4 displays the results of the hierarchical regression where group was added at step 1, child behaviour at step two and avoidant coping at step 3. The first model, with group as the independent variable, was significant, *F* (1, 169) = 4.498, *p* = 0.035, SE = 0.109. The addition of child behaviour scores in model 2 explained an additional 18% of variance in caregiver depression, after controlling for group, Δ *R* ^2^ = 0.18, *F* change = 37.024 (2, 168), *P* < 0.001, SE = 0.068. In the final model, avoidant coping was statistically significant, *F* change = 42.055 (1, 169), *p* < 0.001, SE = 0.099. The final model explains 36% of the variance in caregiver depression (*R* ^2^ = 0.36).

#### Time 2

##### Caregiver stress

Table 5 displays the results of the hierarchical regression where group was added at step 1, child behaviour at step two and avoidant coping at step 3. In model one, group did not explain significant variance in caregiver stress, *F* (1, 107) = 3.232, *p* = 0.075, SE = 0.209. The addition of child behaviour scores in model 2 explained an additional 10% of variance in caregiver stress, after controlling for group, Δ *R* ^2^ = 0.10, *F* change = 12.734 (2, 106), *p* < 0.001, SE = 0.086. In the final model, only avoidant coping was statistically significant, *F* change = 13.011 (1, 107), *p* < 0.001, SE = 0.224. The final model explains 23% of the variance in caregiver stress *R* ^2^ = 0.23). Table 5 also shows the next significant model with emotion focussed coping being added at Step 3, *F* (1, 107) = 16.503, *p* < 0.000. This model explains 7.4% of the variance (Adjusted *R* ^2^ = 0.074).

**TABLE 5:** Hierarchical regression summary: Caregiver stress, anxiety and depression at time 2.

			95% CI					
Outcome	Variable	B	LL	UL	SE B	β	*R* ^2^	(Δ) *R* ^2^
Caregiver	*Step1*							
Stress	Constant	2.30	1.63	2.97	0.34			
	Group	0.38	−0.04	0.80	0.21	0.17	0.03	0.03
	*Step 2*							
	Constant	1.76	1.06	2.46	0.35			
	Group	−0.03	−0.48	0.43	0.23	−0.01		
	DBC	0.31	0.14	0.48	0.09	0.37	1.33	0.10
	*Step 3 for AC*							
	Constant	−0.86	−2.45	0.73	0.80			
	Group	0.06	−0.37	0.50	0.22	0.03		
	DBC	0.17	−0.01	0.35	0.09	0.21		
	AC	0.81	0.36	1.25	0.22	0.81	0.23	0.10
	*Step 3 for EFC*							
	Constant	0.49	−0.63	1.61	0.57			
	Group	0.11	−0.34	0.56	0.23	0.05		
	DBC	0.21	0.03	0.39	0.09	0.26		
	EFC	0.05	0.16	0.09	0.02	0.27	0.20	0.06
Caregiver	*Step1*							
Anxiety	Constant	1.30	0.53	2.06	0.39			
	Group	0.47	−0.01	0.94	0.24	0.19	0.03	0.03
	*Step 2*							
	Constant	0.47	−0.29	1.24	0.39			
	Group	−0.15	−0.64	0.34	0.25	−0.06		
	DBC	0.47	0.28	0.65	0.09	0.50	0.22	0.19
	*Step 3 for AC*							
	Constant	−1.68	−3.46	0.09	0.90			
	Group	−0.08	−0.56	0.41	0.24	−0.03		
	DBC	0.36	0.16	0.56	0.10	0.38		
	AC	0.66	0.17	1.16	0.25	0.25	0.27	0.05
	*Step 3 for EFC*							
	Constant	−0.79	−2.02	0.44	0.62			
	Group	−0.01	−0.50	0.48	0.25	−0.01		
	DBC	0.37	0.18	0.57	0.10	0.40		
	EFC	0.05	0.01	0.10	0.02	0.23	0.27	0.05
Caregiver	*Step1*							
Depression	Constant	1.77	1.05	2.48	0.36			
	Group	0.42	−0.02	0.87	0.22	0.18	0.03	0.03
	*Step 2*							
	Constant	1.26	0.50	2.02	0.38			
	Group	0.04	−0.45	0.53	0.25	0.02		
	DBC	0.29	0.11	0.48	0.09	0.33	0.11	0.08
	*Step 3*							
	Constant	−2.05	−3.74	−0.37	0.85			
	Group	0.15	−0.31	0.61	0.23	0.06		
	DBC	0.12	−0.06	0.31	0.09	0.14		
	AC	1.02	0.55	1.49	0.24	0.40	0.25	0.13

*Note*: Hierarchical model was carried out, group was entered as a dichotomous variable at step 1, child behaviours that challenge (DBC scores) at step 2 and coping strategy—avoidant coping and emotion focussed coping at step 3 with caregiver stress, anxiety and depression as the outcomes.

Abbreviations: AC, avoidant coping subscale; CI, Confidence interval; DBC, Developmental Behaviour Checklist; EFC, Emotion Focussed Coping; IL, upper limit; LL, lower limit.

##### Caregiver anxiety

Table 5 displays the results of the hierarchical regression where group was added at step 1, child behaviour at step two and avoidant coping at step 3. In model 1, group did not explain significant variance in caregiver anxiety, *F* (1, 107) = 3.820, *p* = 0.053, SE = 0.240. The addition of child behaviour scores in model 2 explained an additional 19% of variance in caregiver anxiety, after controlling for group, Δ *R* ^2^ = 0.19, *F* change = 25.316 (2, 106), *p* < 0.001, SE = 0.093. In the final model, avoidant coping was statistically significant, *F* change = 7.024 (1, 105), *p* = 0.009, SE = 0.250. The final model explains 27% of the variance in caregiver anxiety *R* ^2^ = 0.27). Table 5 also shows that another significant model emerged when emotion focussed coping was added at Step 3 F (1, 105) = 6.609, *p* = 0.012, SE = 0.021, explaining 27% of the variance (*R* ^2^ = 0.27).

##### Caregiver depression

Table 5 displays the results of the hierarchical regression where group was added at step 1, child behaviour at step two and avoidant coping at step 3. The first model, with group as independent predictor, was not significant, *F* (1, 107) = 3.558, *p* = 0.062, SE = 0.224. The addition of child behaviour scores in model two explained an additional 8% of variance in caregiver depression, after controlling for group, Δ *R* ^2^ = 0.08, *F* change = 9.831 (2, 106), *p* < 0.001, SE = 0.093. In the final model avoidant coping was statistically significant, *F* change = 18.415, *p* < 0.001, SE = 0.237. The final model explains 25% of the variance in caregiver depression *R* ^2^ = 0.25).

## DISCUSSION

The current study is the first study to look at both child challenging behaviours and caregiver distress in families with and without children who have intellectual disability during and after lockdown in the UK. It was predicted that socio‐demographic factors such as caregiver education level and employment status would be related to caregiver coping, stress, anxiety and depression; however, there were no significant associations between demographics, caregiver coping and caregiver psychological distress. This finding contrasts with research which found that caregivers who are younger, or on lower incomes reported increased psychological distress during COVID‐19 (Beach et al., 2021; Russell, et al., 2020). The divergence in results may be a related to the stage of COVID‐19 lockdown, when data was collected, as well as the country. Beach et al. (2021) and Russell et al. (2020) were carried out at an earlier stage of the COVID‐19 pandemic in the United States. Further research is needed to better understand the role of demographics on caregiver coping and distress across different countries throughout the different COVID‐19 transition periods.

It was predicted that higher levels of avoidant, problem focussed and emotion focussed coping, would be observed during lockdown compared to after lockdown. This was supported since there was a significant effect of timepoint indicating that caregivers showed higher levels of all coping behaviours during Covid‐19 (T1) compared to after Covid‐19 (T2). Paired samples *t*‐tests also showed that caregivers engaged in avoidant coping least and engaged in more emotion focussed coping. As mentioned previously, avoidant coping and emotion focussed coping have been associated with poorer mental health outcomes (e.g. Macdonald, 2011), leading to the tentative conclusion that coping strategies may be a useful area to target for to help support caregivers during difficult times. Covid‐19 timepoint was also found to interact with problem focussed coping only with caregivers reporting higher levels of this coping during T1 compared to T2. This is often reported as an adaptive coping strategy that can lead to better wellbeing in parents regardless of child behaviours (Smith et al., 2008) so it was encouraging to see that the current sample of caregivers defaulted to this strategy during the intense period of Covid‐19 lockdown.

Another hypothesis was that psychological distress would be higher in all caregivers during Covid‐19 (T1) compared to after Covid‐19 (T2). This was supported and aligns with findings from other countries, for example, Johnson et al., 2022 reported increased levels of caregiver distress in Norway during lockdown/school closure periods. All caregivers in the current study also showed significantly higher levels of stress and depression compared to anxiety. Depression specifically in caregivers during Covid‐19 has been found in other studies (Gallagher & Wetherell, 2020) and may be associated with the high levels of loneliness experienced during the pandemic. Lack of social support and socialising opportunities may lead to intense periods of being with the family with little respite, and this may have also increased caregiver’s perceived burden causing higher levels of depression and stress (Blacher & McIntyre, 2006; McConnell & Savage, 2015). All levels of stress, anxiety and depression were higher for all caregivers during T1 compared to T2 indicating that similar to other Covid‐19 research that caregiver psychological distress was higher during periods of lockdown (Willner et al., 2020).

It is worth noting, that although this study aimed to explore differences between caregivers of children who have and do not have intellectual disabilities, both groups showed similarities in coping strategies during and after Covid‐19. This is an interesting result given the additional barriers and issues that families with children who have intellectual disabilities face (Blacher & McIntyre, 2006; Unwin & Deb, 2011). This may show that the impact of COVID‐19 lockdown was relative to the family context before and during the pandemic and was viewed by all families to increase levels of coping strategies and psychological distress. At time 1, caregivers of children with intellectual disabilities showed higher levels of psychological distress than caregivers of typically developing children. This aligns with previous research indicating that caregivers who have children with more complex needs experienced higher levels of distress during the pandemic (Iovino et al., 2021; Willner et al., 2020) and supports the notion that this is a vulnerable group who are more at risk of negative mental health outcomes.

It was proposed that child behaviours that challenge would be significantly higher during lockdown compared to after lockdown. This was indeed supported, although comparison of T1 to T2 data must be interpreted cautiously due to the attrition rate. However, this finding does align with other cross‐sectional research carried out in other countries such as China (Chen et al., 2021) which found significantly higher child mental health difficulties and behaviours that challenge during periods of school suspension. Relatedly, it was also found that parents of children who have intellectual disabilities reported higher levels of behaviours that challenge. This makes sense since intellectual disabilities are often characterised by adaptive, cognitive and social skill difficulties (Zayac & Johnston, 2008) which are associated with behaviours that challenge (Lee, et al., 2008) impacting on caregiver stress (McConnell & Savage, 2015).

It was also predicted that higher levels of child behaviours that challenge would be related to caregiver distress during both time points (T1: during lockdown and T2: after lockdown). Higher levels of child behaviours were found to be related to higher levels of caregiver stress, anxiety and depression in both caregiver groups (caregivers who have children with and without intellectual disability) at T1. Higher levels of child behaviours that challenge were related to higher levels of caregiver anxiety in both caregiver groups at T2, which is in line with previous research reporting the interdependent relationship between child behaviours that challenge and caregiver mental health outcomes (Baker et al., 2003; Blacher & McIntyre, 2006; Floyd & Gallagher, 1997). The children’s levels of behaviours being related to more caregiver psychological distress at T1 may have been caused by the increased time spent together within close proximity. The easing of lockdown may have allowed more time to be spent separately either by returning to the workplace, school, or engaging in more lone leisure time, reducing levels of distress in caregivers and children’ behaviours that challenge.

It was further proposed that child behaviours would be related to higher levels of all caregiver coping strategies (avoidant, problem focussed and emotion focussed). In the typically developing group child behaviours that challenge were related to avoidant coping (e.g. self‐distraction, denial, behavioural disengagement) during T1 and both avoidant and emotion focussed coping (e.g. venting, use of emotional support, humour) during T2. For the caregivers of children with intellectual disability, child behaviour was related to both avoidant and emotion focussed coping at T1 and avoidant coping at T2. This is similar to other research such as Petrocchi et al. (2020) who found relationships between mothers’ coping abilities during COVID‐19 lockdown and children’s adaptive behaviours through the mediation of child positive emotions. Although the Petrocchi et al. (2020) study demonstrates a similar relationship in the opposite direction compared to the current study findings. Taken together, both studies show a complex bi‐directional relationship between child behaviours and caregiver coping that needs to be considered in all coping research.

It was also predicted that higher levels of avoidant and emotion focussed coping and lower levels of problem focussed coping would be associated with higher levels of stress, anxiety and depression during both time periods. Child behaviours that challenge and caregiver coping was shown to be significantly related to psychological distress in both groups at both time points. Child behaviours were found to be significantly related to all psychological distress outcome measures (stress, anxiety and depression) at both time points. Additionally, it was found that levels of avoidant coping and emotion focussed coping was associated with caregiver stress and anxiety in both caregiver groups in T1 and T2. Only avoidant coping was associated with depression at both time points. The regressions also showed that avoidant coping explained higher levels of variance with stress, anxiety and depression at two time points indicating that this coping strategy may be an important catalyst in caregiver psychological distress. Interestingly, this is both similar and different to research carried out by Fluharty et al. (2021) in the general UK population during the first 21 weeks of lockdown. They found that avoidant coping predicted poorer mental health outcomes (depression and anxiety) and emotion focussed coping led to improved outcomes which deviates from the current study findings that high levels of both coping types were associated with psychological distress. These slight differences might have been caused by the current study focussing on psychological distress measures as opposed to mental health outcomes or it may indicate that as lockdown continued some coping strategies that were adaptive to start with became maladaptive over the long term.

Interestingly, problem‐focused coping did not explain significant variance in psychological distress. This does not align with other research carried out on caregivers, for example, Smith et al. (2008) found that problem focussed coping was associated with better maternal wellbeing. This difference may be because the current study focussed on psychological distress as opposed to wellbeing and may indicate that coping is associated with distress and wellbeing differently. In addition, earlier analysis also showed that problem focussed coping was the only coping strategy that significantly reduced in T2 compared to T1. A potential explanation, since problem‐focused coping refers to ‘actively seeking solutions to problems’, is that this is a more prominent strategy to use during intense periods of stress such as lockdown. The lack of association between problem focussed coping and distress is different from previous research which has found that this type of coping reduces poor psychological outcomes in adults (for a review, see Penley et al., 2002) and might suggest that problem focussed coping is not related to poor psychological outcomes in caregiver groups specifically. Caregivers in the current study seem to only use problem focussed coping as a strategy during intense and stressful periods and that this strategy did not associate with their distress. More research is needed to further explore which coping strategies are used by different participant groups and how these strategies impact distress and wellbeing.

### Limitations

This study had some limitations. Firstly, the current study relied on caregiver‐reported diagnoses and behaviours which may have caused some bias in reporting; although, previous research has indicated that parent‐reports are relatively reliable and robust (Rosenberg et al., 2009). Secondly, in the intellectual disabilities group, there was a range of developmental diagnoses, meaning different child characteristics could have influenced the intensity of child behaviours, to which caregivers were exposed (McClintock et al., 2003) ultimately impacting psychological distress outcomes. Therefore, future studies should examine differential pathways exist relative to specific diagnoses. Finally, as noted earlier, there was a substantial attrition rate between time 1 and time 2 (36.3%). Although no systematic differences were found between those who completed timepoint 2 and those who dropped out, no assumptions can be made about the statistical behaviour of those who dropped out. Group differences must therefore be interpreted with caution.

### Conclusions

Despite these limitations the current study offers an insight into behaviours that challenge in children with and without intellectual disability and their caregivers’ mental health during the COVID‐19 lockdown at different time points in the UK. The study shows that both child behaviours and caregiver psychological distress appeared to reduce as lockdown ended; however, it is possible that caregivers’ coping strategies may have contributed to increased mental health symptoms. As coping behaviours could be effectively targeted during times of stress to reduce poor mental health outcomes, tentative implications can be drawn for supporting caregivers during periods of high stress.

## AUTHOR CONTRIBUTIONS

**Karri Gillespie‐Smith**: Conceptualization; Formal analysis; Investigation; Methodology; Project administration; Supervision; Writing original draft; Writing review & editing. **Karen Goodall**: Conceptualization; Formal analysis; Investigation; Methodology; Writing original draft; Writing review & editing. **Doug McConachie**: Conceptualization; Investigation; Methodology; Writing original draft; Writing review & editing. **Jo Van Herwegen**: Conceptualization; Investigation; Methodology; Writing review & editing. **Hayley Crawford**: Conceptualization; Investigation; Writing review & editing. **Carrie Ballantyne**: Conceptualization; Investigation; Methodology; Writing review & editing. **Caroline Richards**: Conceptualization; Investigation; Writing review & editing. **Tom Gallagher‐Mitchell**: Conceptualization; Investigation; Methodology; Writing review & editing. **Jo Moss**: Conceptualization; Investigation; Writing review & editing. **Grace Khawam**: Data curation; Investigation; Writing review & editing.

## CONFLICT OF INTEREST STATEMENT

All authors disclosed no relevant conflict.

## ETHICAL CONSIDERATIONS

The data used in this study were collected with ethics approval granted by the Department of Clinical and Health Psychology Research Ethics Committee at the University of Edinburgh (reference number Ethics070).

## Data Availability

Data currently available on request from the authors however will be openly available after 31st August 2024 on Edinburgh DataShare—https://datashare.ed.ac.uk/.
